# Operating Ethnicity-Focused Senior Long-Term Care Homes in Ontario, Canada During the COVID-19 Pandemic

**DOI:** 10.3390/ijerph23020152

**Published:** 2026-01-26

**Authors:** Anukrati Nigam, Robert Chin-See, Kirolos Nour, Akshaya Neil Arya

**Affiliations:** 1 Collaborative Program in Neuroscience, Institute of Medical Science, Temerty Faculty of Medicine, University of Toronto, Toronto, ON M5S 3K3, Canada; 2 SoundLife Scarborough, Department of Arts, Culture, and Media, University of Toronto Scarborough, Toronto, ON M1C 1A4, Canada; 3 Association for Socially Applicable Research, Pune 411007, Maharashtra, India; 4 Department of Health Sciences, Wilfrid Laurier University, Waterloo, ON N2L 3C5, Canadanour6240@mylaurier.ca (K.N.); 5 Department of Family Medicine, McMaster University, Hamilton, ON L8P 1H6, Canada; 6 School of Public Health Sciences, University of Waterloo, Waterloo, ON N2L 3G1, Canada

**Keywords:** ethnicity, long-term care, cultural safety, immigrant, ageing, COVID-19 pandemic

## Abstract

**Highlights:**

**Public health relevance—How does this work relate to a public health issue?**

- Highlights the systemic challenges within LTC settings during a public health emergency.
- Examines how the decision makers, managers, and leaders of LTC homes catering to vulnerable groups disproportionately impacted during the COVID-19 pandemic in Ontario, Canada, faced the COVID-19 pandemic.

**Public health significance—Why is this work of significance to public health?**

- Identifies how pre-existing systemic and health inequities, cultural and language barriers, and funding limitations intensified vulnerabilities among elderly LTC residents during COVID-19.
- Analyzes perspectives of decision makers, managers, and leaders of ethnicity-focused senior LTC homes during the COVID-19 pandemic, groups often underrepresented in the literature, adding depth to the existing literature on COVID-19 pandemic impacts and preparedness.

**Public health implications—What are the key implications or messages for practitioners, policy makers, and/or researchers in public health?**

- There is a need for integration of culturally responsive, people-centred approaches into pandemic preparedness, funding models, staffing strategies, and infection prevention and control planning for LTC homes.
- There is a need to prioritize construction and management of LTC homes, considering the special needs of ageing and diverse populations in the Global North.

**Abstract:**

Canada’s ageing population continues to grow rapidly and significantly more diverse, which will require unique health and home service needs. The COVID-19 pandemic exacerbated existing challenges in Canada’s healthcare system and demonstrated the need for long-term care (LTC). Semi-structured interviews were conducted with 17 decision makers, managers, and leaders in long-term ethnically focused facility care. Braun & Clarke’s six-stage process of thematic analysis was applied using an iterative, deductive approach to examine the experiences of stakeholders involved in the operational, managerial, financial, and clinical aspects of an ethnicity-focused LTC. Findings highlighted salient characteristics of impactful ethnicity-focused care and factors were uniquely shaped by the delivery of culturally specific care. Key subthemes included social isolation and emotional impact, operational and logistic difficulties during COVID-19, mitigation measures implemented in response, and the social, behavioural, and health benefits observed among seniors living in these LTC homes. Participants identified political and economic constraints (e.g., provincial funding) to establishing ethnicity-focused care homes but proposed several solutions and highlighted potential benefits that could support successful implementation. Analysis of experiences of operational challenges in safely and adequately running ethnicity-focused LTC reinforces the value of ethnicity-focused LTC during times of crisis such as the COVID-19 pandemic, as they provide a culturally safe and familiar space with preventive measures applied in a timely manner for seniors to engage with their peers in an environment that meets their health needs, ensuring safety standards.

## 1. Introduction

Canada is a nation of newcomers where almost a quarter of the population in 2021 (nearly 23%, ~8.3 million people) was foreign-born [1]. The ageing population in Canada, as of July 2025, was at ~8.1 million [2], contributing to 19.5% of the entire population [3]. According to Employment and Social Development Canada, 30% of the total senior population in Canada is composed of immigrants [4]. Thus, Sagbakken et al. highlight the needs of caring services and socio-cultural programming to reduce isolation and linguistic barriers [5]. An environmental scan by Hsu et al. underscored the challenges of seniors identified as official language minorities living in long-term care (LTC) homes in Canada [6]. They reported issues with translation and a need for language provisions in healthcare and cultural programming, and the linguistic needs of seniors in LTC homes often remain unmet. Through focus group discussions, Cuevas et al. found disparities in patient–provider relationships among African American, Latin American, and European American patients [7]. In particular, Latin American patients preferred language/culturally concordant clinicians and African American patients perceived discrimination from providers. Such disparities can persist in long-term ethnicity-focused care homes and have disproportionate impacts during a global pandemic.

On 11 March 2020, the World Health Organization declared a global pandemic involving the novel coronavirus (COVID-19) [8]. Within a week, provincial and territorial authorities in Canada declared a state of emergency. LTC homes were one of the hardest-hit sectors, with localized outbreaks occurring in Canada, with higher mortality rates in populations aged 70 years or older [8,9,10]. When attention was drawn to LTC systemic issues, with tragedies in Quebec resulting in the summoning of the army early during the COVID crisis, Béland and Marier stressed the need for LTC policy to be prioritized in the social policy agenda of decision makers in Canada [11]. In a preliminary survey analysis of Canadian LTC homes, Clarke underlined absenteeism among the staff, along with increased needs related to safety measures (handwashing and usage of personal protective equipment (PPE)) as key challenges during the COVID-19 pandemic period [12]. Outbreaks in nursing homes were attributed to shared rooms, large facilities, and infection cases among employees. Guttmann et al. reported that over 43.5% of the COVID-19 cases in Ontario were among immigrants, refugees, and newcomers, which compose 25% of its population [13]. Systemic barriers exacerbated these cases of racialized populations working in LTC homes [14]. The Congregate Care Setting Working Group and the Ontario COVID-19 Science Advisory Table identified five key areas for improving the experience of LTC home residents: improvement in staff working conditions, shift towards private rooms, developing infection prevention and control expertise within homes, access of essential caregivers to residents, and timely and high-quality palliative care to residents [15].

In addition to the implementation of infection prevention and control protocols and measures, Fahim et al. outlined other challenges, including support for COVID-19 vaccine access and confidence and concerns regarding well-being due to COVID-19 pressure in LTC homes and retirement homes [16]. Estabrooks et al. found growing clinical complexity in chronic conditions and comorbidities among residents, lack of adequate training of, and support to, the workforce, and lack of integration of LTC homes with healthcare centres [17]. Studies by Deber et al. analyzed LTC homes in Ontario, Quebec, and the Netherlands [18], and studies by Grinspun et al. involved comparison of LTC homes in four countries—Australia, Canada, Spain, and the USA [19]—found that a broader approach was deemed necessary, including policy reforms. The latter structured evidence-based interventions at the macro, meso, and micro levels. The macro level focused on improving funding, transparency, accountability, and health system integration and promotion for not-for-profit and government-run LTC facilities. The meso level included structural change towards “green houses” with fewer than 20 residents and private bathrooms instead of a warehouse design. The micro level included staff management and training, preventive measures for infectious diseases, and mental health and well-being support, among others.

In this study, we explored and reported findings on the needs of ethnicity-focused care homes in Canada for the ageing multicultural and linguistically diverse population in emergency planning settings. Semi-structured interviews with 17 managers, staff, and policy makers involved in the LTC of seniors highlighted the challenges faced by ethnicity-focused senior homes during the COVID-19 pandemic and the needs and opportunities that arose to improve service in these LTC homes. We use the term ethnicity-focused senior LTC homes to describe the different LTC homes that cater to different ethnicities in the multi-ethnic population of Ontario, Canada, based on their cultural, linguistic, and social needs.

## 2. Methods

The study began just prior to the onset of the COVID-19 pandemic in Canada, where CG began gathering data at the beginning of March 2020 (prior to the declaration of the COVID-19 pandemic in March 2020) and continued during this period. While not initially focused on COVID-19, it evolved early during data collection to include questions related to its impact. Four members of the research team (CG, RCS, KN, and SA) conducted the interviews, including a clinician, a medical trainee, and undergraduate students. A qualitative approach was chosen to enable open and in-depth exploration of participant experiences. This study was approved by the Research Ethics Board at Wilfrid Laurier University (REB #6467), and all participants provided verbal or written consent.

### 2.1. Recruitment

Potential participants were shortlisted from initial scoping news searches and grey literature regarding ethnically focused LTC homes and thereafter with snowballing, based on suggestions from initial informants who were primarily the managers of LTC homes interviewed for this study. Though we intended to include contacts within the LTC sector, such as staff, policymakers, managers, directors, etc., in the end, managers and leaders in ethnically focused LTC were included in this study. The majority of participants were enlisted based on a search for culturally sensitive homes in the Greater Toronto Area (GTA), particularly seeking appropriate management or staff members of these facilities. These ethnic LTC facilities varied in the number of residents who could be accommodated and catered to different minority populations, primarily of Asian and European ancestries. More detail is not provided to protect the identities of these individuals, as this is a sector with few facilities and fewer leaders managing each facility. These individuals were then approached through email exchanges, telephone messages, or both. Those who expressed interest in participating in the study were then sent information about data confidentiality, study withdrawal, and consent, which they had to return prior to the interview. Three further attempts to contact them were made, unless participants expressed disinterest or declined. The 17 interviewees participating in the study reflect a spectrum of COVID-19 pandemic experiences from 10 or more ethnic LTC homes in the GTA region.

### 2.2. Data Collection

Data was collected based on our semi-structured interview questions designed to address a number of key components to understanding the feasibility of developing and implementing ethnicity-specific long-term care facilities. These elements were drawn from both the PI’s (ANA) experience in primary care with a multicultural context, including in the LTC sector, as well as from the literature on cultural LTC. Broadly, the interview guide explored key organizational, logistical, and contextual factors that influence the management of ethnicity-focused care homes and factors that shape the health and well-being of residents. Audio recordings of the interviews were transcribed using Otter transcription. SA, CG, and RCS conducted the interviews, and AN, RCS, and KN assisted with coding. CT provided advice and training regarding qualitative analysis.

### 2.3. Data Analysis

The analysis employed structured coding procedures and thematic characterization of textual data obtained from interviews (Supplementary File S1). Data was approached iteratively through Braun and Clarke’s (2006) six-stage process of thematic analysis to identify emergent themes consistent with objectives and participant experiences [20]. Briefly, the framework entails (1) familiarization with data, (2) generating initial codes, (3) searching for themes, (4) reviewing themes, (5) defining and naming themes, and (6) writing the report. In the initial phase, to reduce analytical biases, a team member who was not involved in the interview process (AN) carefully reviewed all interview transcripts and became familiarized with the data. Insights from the existing literature were integrated to contextualize findings and enhance analytical depth, resulting in a hybrid strategy that facilitated the identification of salient cultural and systemic barriers. A principal codebook was constructed after preliminary analysis of interview transcripts through an inductive approach. Researchers (AN, RCS, and KN) meticulously analyzed the transcripts and independently executed initial coding to develop the codebook. The coding framework was amended through iterative discussions.

## 3. Results

**Figure 1:**
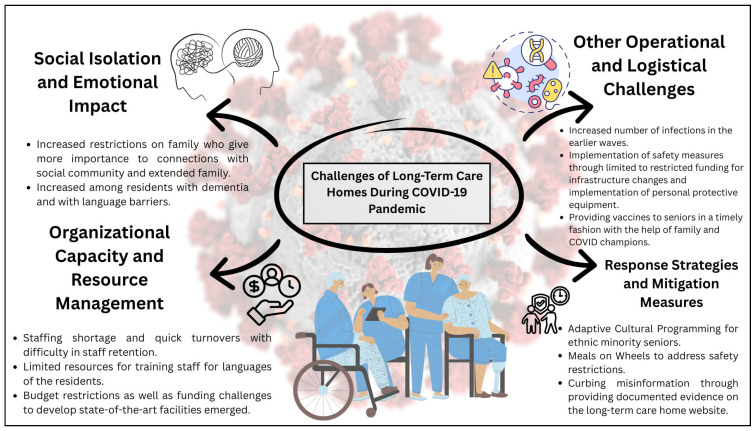
Challenges of ethnicity-based LTC homes during COVID-19 pandemic, identified as per thematic analysis from semi-structured interviews of managers of LTC homes in Ontario, Canada. Four themes were identified—Social Isolation and Emotional Impact, Organizational Capacity and Resource Management, Other Operational and Logistical Challenges, and Response Strategies and Mitigation Measures—which encompass the challenges faced and addressed by the managers of LTC homes during the COVID-19 pandemic.

The COVID-19 pandemic presented an unprecedented challenge for ethnicity-focused senior care facilities in Ontario. We observed four subthemes from the thematic analysis of the interviews with managers of ethnic senior homes in Ontario, Canada. Key themes of Social Isolation and Emotional Impact, Organizational Capacity and Resource Management, Other Operational and Logistical Challenges, and Response Strategies and Mitigation Measures were identified. Figure 1 showcases the key themes and subthemes identified from the analysis of the semi-structured interviews with managers of LTC homes. In amalgamating the results, we highlight the key points and quotes from the interviewees. In some cases, our interviewees’ first language was not English, and we have chosen to be faithful to the language that they employed, with minor grammatical corrections, unless clarity was affected.

### 3.1. Social Isolation and Emotional Impact

One of the biggest challenges of the COVID-19 pandemic was the physical and mental health of individuals. Social isolation due to the implementation of quarantine and preventive measures had a profound impact on the mental health of LTC residents. Uncertainty about the evolving situation due to the pandemic led to limited family and staff interaction, which caused an emotional impact on the residents.

Interviewee #008 mentioned that the impact was “*a lot because of the additional practices that we’ve put in place, and you know, all those infection control, things like that. I think one of the biggest effects that COVID has had that we really cannot measure is the mental effects and the psychological effects that have affected the seniors because they can’t, couldn’t have their family members here. Like visits, because often, it is the family that is their kind of lifeline for the language and everything. So, from that perspective, it was least for the residents who were cognitively aware and could appreciate that there’s been a change and it’s very hard to determine for the residents who are cognitively impaired what they were really feeling and experiencing because it’s very hard to really make that determination.*”

These restrictions extended to include the frequency of visits, which was a challenge for families who placed more importance on social connections and the extended family of residents. Interviewee #008 stated, in this regard, “*Well part of the visitation, certainly because of the COVID. I mean, at one point, we weren’t allowing family givers to come in at all. Then it was, an hour that essential caregivers can come in. So again, they’re not seeing all their family members. Maybe they had kids that dropped in once a month.*” Interviewee #008 also addressed spatial constraints: “*That’s right, and we just don’t have that physical space to look at doing anything like that. For our residents, when the pandemic first started, they were basically without a word of a lie, they were basically confined to their rooms. There was no way that we could even bring them out into a lounge area in small groups to at least have a change of scenery or something.*”

Interviewee #006 explained the direct impact on mental health and well-being when patients could receive visitors—“*So I mean on one hand, like there is risk long-term care homes are under siege by the virus right now. And then on top of that, no one can see what’s going on. Like no one can go in and touch their grandma or no one can give their grandpa a hug. They can’t. It’s not this crazy convergence of awful circumstances. It means that people are at least able to address their most pressing concerns.”* Interviewee #004: *“What I will say there will be much influence in terms of providing care through a telephone, as well as on an online platform. …we know that social isolation is a very important issue in the older adult population. And especially around this time, they can’t go anywhere, well not even grocery shopping is not recommended. They can’t go to community centers and things like that. And for long-term care residents, they can have their family members come to visit them, only as [an] essential visit…. So, during this time, I would say that the emotional support as in, as well as the mental support would be very critical.”*

Interviewee #011 observed cultural differences playing a role in the extent of connectivity expected in the long-term care homes. Certain minority cultural groups who were used to living in multigenerational setups where elders are respected and not to be abandoned were more vulnerable to the separation and isolation restrictions. Interviewee #011 mentioned, “*And again, acknowledging the risk of stereotyping. North American, or North American western culture, generally do not put as much value, but they do not put as much emphasis on multi-generational conductivity. They value the visit by children or grandchildren. But they do expect children to grow and live their own lives. And then the older persons, as much as possible, have their own independence and choose their own life, even when they go into long term care. But the expectation of some minority cultural groups would be very much in terms of filial piety in the sense that there is a respectful elder and that they are not to be abandoned by the younger generation. So, they would expect to enforce separation isolation, perhaps a bit more acutely.*”

Because of these particular limitations on social interactions, we observed interviewees sharing the emotional impact of the COVID-19 pandemic. Feelings of vulnerability were notably heightened between family members and residents due to visitor bans in resident homes. Interviewee #006 spoke of the emotional impact and of observing a state of confusion, uncertainty, and vulnerability—“*We were preparing for this pandemic intensively since December, but then in March … when nothing was familiar. The very first thing that our families would have seen was a visitor ban and that was an essential step to keep the facilities safe. I don’t contest the ministry decision to do that, but what it meant was at exactly the moment when our families were most vulnerable, the curtain came down and, and it was no longer possible to see what was going on inside. …I deduce that our families were, and are, terrified for the safety of the elders living in our long-term care facilities.*”

### 3.2. Organizational Capacity and Resource Management

Organizational Capacity and Resource Management comprises human resources such as staffing (hiring, shortage, and limited availability) and financial barriers such as the availability of funds for additional costs to ensure consistent programming. The closing of ethnically focused care homes due to budget restrictions, as well as funding challenges to develop state-of-the-art facilities, emerged. Interviewee #006 shared. “*The religious stuff is suspended… and these are all the building blocks of a culturally appropriate experience. Yeah, they’re essential.*” Interviewee #012 noted additional expenses: “*Financially it affected us because we just spray the place twice a day. We’ve tried to make sure that they wear a mask, we got their vaccines to them.*”

During the COVID-19 pandemic, management faced difficulties in hiring, as the turnover rate was high, especially for nurses. New staff, even when found, were less likely to be familiar with the clients’ maternal tongues, and it was harder to be more particular about meeting these needs, given the labour shortage. Facilities that experienced higher morbidity and mortality faced greater challenges in replacing and training new staff. Interviewee #015 spoke in this regard—“*COVID-19 was very devastating for this facility. We lost a lot of people in the first year, recovered well in the second year, but immediately we had to close down all these wonderful things I just spoke about … halls got closed down, services got shut down. because we’re in [hard hit city], a lot of staff got COVID. So, we had to bring in a lot of agency staff and you bring agency staff, it means it’s people they don’t know, they’re not familiar with them.*” Interviewee #013 explained, “*We all know that it’s hard to hire nurses now… So obviously, turnover is a big problem. So, when we hire quickly, and sometimes we’re not able to hire someone with the right background to serve our residents.*”. The staffing shortage extended to volunteers who had worked with cultural programming and often knew the clients’ languages, but who were unable to continue working due to safety restrictions. Interviewee #017 shared, “*While it affected us and still is affecting us, because first of all, we lost almost all of our volunteers. And then we’ve been short staffed ever since it’s a constant challenge even. We have people that come in, and they will, if they don’t like what’s going on, or they don’t think they get offered more money elsewhere, especially for that they’ve got to work for an agency because they can pick and choose their locations and the days they want to work and make their own schedule, basically following the line from us*.”

With challenges also came opportunities. Interviewee #001 shared, in this regard, “*Well, except for now, now we will, we were one of the first to close, because we recognized that we’ve become a vector for illness. Happily, no staff or clients became ill. But the fact that these people were already compromised and working in these areas was just mind boggling…Fortunately, one of the silver linings I think will come out of COVID is a push to build state-of-the-art, infection, controllable, long-term care.*”

A critical aspect that was observed from the managers’ interviews was the challenges of caregiving and running an ethnicity-focused senior home in the Ontario region in Canada. Interviewee #014 mentioned that language and communication challenges arose as information about COVID-19 and its updates could not be solely disseminated in English. “*Well, I think that COVID-19 has impacted hard, [we] need to really increase our communication. So, we’re needing to ensure that all of our people living in our homes have the proper communication. We primarily deliver our communications in English at the corporate level, but then when it comes time to actually talking to the residents or family members. That might be in the person’s specific language to help them understand what is happening. And so, we’ve had to be really mindful of communication and what everyone needs in order to receive the proper information and ensure that everyone is well.*”

Interviewee #015 noted the importance of relationship building with sponsors, advocacy, and maintaining political connections to deal with extreme delays in funding of more than 18 months. “*They (the Ministry) pretend to have funded COVID. But they don’t give you the money… [till] more than it’s like a year and a half later. I had to make some calls about that. Because I said, come on, we’ve got to pay our bills. And we’ve got this receivable that the Ministry of Health has put forth.*”

Technological challenges became prominent in long-term care homes, where residents had to rely on virtual appointments and were unfamiliar with virtual video conferencing platforms and the transition to call centres. Interviewee #016 relayed, “…*A lot has changed them, where they had a caseworker, and now it’s sort of even, for example, Public Guardian trustee. Clients would have their trustee’s phone number, now, it’s all a call center. And it takes forever. And many of my clients don’t have the capacity to navigate.*” Involvement of stakeholders and policymakers ensured an emphasis on coordination. Interviewee #017 disclosed, “*And we were working with our Ontario Health Team and we’re working very closely with the Sunnybrook Hospital and on our infection control practices and everything and a lot of emphasis on that, so right now everybody gets screened and gets tested and so forth*.”

The managers of multicultural long-term care homes faced challenges in implementing fairness as a principle so that a way forward could be mapped in considering the ethnicities of the resident population and evolving together. Interviewee #011 shared, “*Perhaps it reflects my professional background, individuation and personalization and client sentiments is always to go by, whether you’re talking about working with a particular community or cultural group, then what is your understanding of the cultural group? How are you going to leverage the strength of that particular ethnic cultural group and their values and evolve your way of working with them? Understanding that multiculturalism is an ideal state, it does not guarantee that your whole facility, your whole staff group or the rest of your clients, shares in that viewpoint in the same way, and so be careful about what would be seen as favoritism to particular groups. What is fair and accommodating?*”

### 3.3. Other Operational and Logistical Challenges

The COVID-19 pandemic resulted in operational and logistical challenges for ethnically focused LTC homes, which resulted in increased direct and indirect costs due to infrastructure, quarantine challenges, and implementation of personal protective equipment measures mandated by governing agencies.

Interviewee #013 disclosed, “*the wings have been made into an isolation wing. Unwillingness now, or was previously during the peak of it all, just used to isolate. And then once they’re done isolating [and begin to] move into an actual room. But the isolation rooms were once rooms themselves. So now we can’t work as capacity is lowered? I think that’s the biggest impact as far as space goes*.” Interviewee #013 stated in this regard, “*everything’s more costly, because, for example, shared bathrooms, like no, we have to avoid sharing. We have to avoid admitting patients into rooms that they have to share with others. We’re constructing our long-term care home in [Greater Toronto Area Suburb] now and we’ve had to redesign it a little bit to meet the needs of the new landscape.*”

Interviewees shared experiences surrounding safety issues during the pandemic. They addressed that they continued to face challenges in perceptions of LTC homes among the wider public due to being regularly featured in the news. Infrastructure challenges in implementing safety rules and regulations were prominent due to a lack of physical space to ensure social distancing. As LTC homes initially failed to receive extra funding to address infrastructure challenges, this necessitated budget cuts from other operations. Interviewee #008 observed, “*I really don’t know what we can change because of our physical structure and layout. We don’t have the space to do a lot of things. To build a new building, it would be nice, because then we could incorporate all kinds of things into the new building that would make handling a pandemic, I would think, not easy, but certainly we would be able to manage it in a different manner.* … *because we’re an older building, we don’t have the airflow that would be needed to help sort of keep various sections so that the air isn’t intermixing … So that is a big challenge. And that challenge can never be overcome, until we actually have a brand new physical structure.*” One of the benefits was that tight regulations ensured that safety measures were applied. Further challenges included the wearing of personal protective equipment such as masks, mainly by the staff, and rising expenditures from regular testing. Interviewee #008 remarked, “*Like all the staff are wearing masks. The residents aren’t, but the staff are.*” Rooms where 3–4 beds could be placed initially had their number of beds lowered down to two to adhere to safety regulations (Interviewee #017). Interviewee #017 recalled, “*Well, the one thing that COVID affected us, while you’re well aware of this is, we used to have two, four bed wards. So we’re, we can only have two beds in those rooms anymore.*”

Interviewee #015, who worked in a hard-hit city, shared a concern: “*what’s the biggest challenge was the fact that we are at our staff within [large city], it’s very dead serious, so they get sick. And so all I can do with that really is use PPE, but people are going home to kids who are coming from school. And there’s not a whole lot you could do about the spread of the disease outside of here. Like when they’re in here. They’re [wearing] full gear. When they go home. They’re not. So, I don’t think there’s anything more we could do. I mean, we’re doing the maximum… we’ve gone above public health requirements in terms of testing, and rapid testing, and I’m checking on people. So that’s really all you can do*.” Interviewee #014 explained the importance of relationships: “*certainly, having a close relationship with our resident clients and family members helped. And our staff helps everyone to stay safe. So, because we move together almost like an extended family, and people are more cooperative, right, and they’re more understanding, …. So that all contributes to safety, right, the hand hygiene and asking the vaccinations and when we have new situations come up, they’re very understanding, and we communicate with them quickly, and we resolve their questions. That’s why we’re able to create that environmental safety.*”

Subsequently, the application of vaccines posed a significant challenge in long-term care homes. Often, permission from family members or discussing the benefits of the vaccines with family members was needed to convince residents to be vaccinated, as the medical decisions of the residents were made by their family members. Interviewee #009 shared their experience: “*we did over 400,000 vaccines for seniors in East Toronto. And it was massive. So, bringing them down, calling their daughters, for some of them, I don’t make any decisions, only my daughter does, or all sorts of things. Like, it’s just you have got to the biggest thing we’ve learned from COVID, is you’ve got to meet people where they’re at, not where you think they should be?*”

A novel innovation that might be applied to future public health education activities was the development of COVID vaccine champions who could translate the benefits of vaccines to their own populations. Among some communities stigmatization was noted, for example, resentment of outsiders who advocated for vaccines, and judgment of people who received vaccines. COVID champions could be priests, imams, etc., whom some of the population had more faith and belief in. Interviewee #009 speaks in this regard, “*Part of what we had to do was to translate the information for each of the different communities. But we also then had to find champions in those communities that can help to be ambassadors for that work, so really going to the source and understanding the cultural nuances are really, really important. So, for example, South Asians, like the Indian community, or the Muslim community that was in [region in GTA] and they would listen to their Imams, or their religious leaders, they were the ones who could convince them in another community, it was somebody else. So, I think you have to, and I also worked with a lot of faith-based organizations who were trying to do good work, but doing a horrible job of it, and being able to take because you’re offering them this, but they actually don’t want that.*”

### 3.4. Response Strategies and Mitigation Measures

Response Strategies and Mitigation Measures during the COVID-19 pandemic consisted of policies, innovations, community partnerships, and contingency planning for the residents of ethnically focused LTC homes.

The COVID-19 pandemic negatively impacted the funding received by the ethnicity-focused senior homes. The managers were required to adapt to new cultural programming with pandemic restrictions. This involved switching in-person to virtual programs and ensuring people were not isolated during festivals. There was pressure placed on organizations to rapidly adapt culturally appropriate and other programming to virtual platforms, which was a challenge to accomplish with existing resources, as highlighted by Interviewee #014, who mentioned that “*Well, especially during COVID-19, when we need to pivot so much changing our in person programs to virtual programs, communicating much more frequently when people are isolated and can’t celebrate Christmas or Easter or whatever holiday together, we’re needing to ensure that the meals are extra special, right, that they can also celebrate, even though their loved ones aren’t allowed to come and visit or if you’re just living at home independently, you’re not going out yourself. So, there’s been all sorts of pressures on organizations not only limiting the home, but everywhere, including those who are culturally appropriate to really move fast and just continue to use existing resources without any additional funding to change our programming or to move as fast as we could*.”

Interviewee #004 remarked that an option to comply with safety restrictions due to the pandemic was switching to a Meals on Wheels-based program. Several interviewees highlighted the need for greater resources for online and telephone communication to assure families and the general public during waves of increased illness, while more staffing was needed to care for sicker residents.

Some LTC homes implemented measures focused on disclosure to curb the spread of misinformation, which may have happened more easily in more isolated ethnic minority communities with more limited sources of information, such as tailoring their website through the addition of photos or a separate good news page and updating everyone about the ongoing situation at the ethnic care home. Interviewee #006 commented, in this regard, “*We tried to address that in our communications and this is actually where most of my energy has been focused in the last six weeks. So… on the [ethnic home’s] website., what you’ll find is that our communications are tailored specifically towards disclosure…. we’ll tell you everything that we can…about what’s going on in here. There’s lots of photos. There’s like, we’ve got a whole good news page on our website, which is just about making more visible what it is that we’re doing. If we weren’t doing that, we know that social media would go bananas with all kinds of theories about what was going on inside facilities. But we can proactively address some of that worry and some of that interest, by just showing what we’re up to*.”

Though infection outbreaks in ethnic senior homes did occur, the number of lives lost varied. Interviewee #017 noted, “*And in the beginning, before the vaccines and everything sort of spread, like wildfire. And even, we had a couple of residents that were even bedridden, and they still got COVID. But ever since the vaccines and everything like that, we’ve had some cases, but they don’t get serious illnesses like they did in the beginning of the pandemic.*” After the initial phase, with various measures in place, Interviewee #010 explained, “*So COVID, because we’ve established that community, we didn’t have any problems during COVID time. We just treat everyone the same way. And, we’ve never had other than one outbreak with one resident that made it, die of it. And it was actually a year, we’ve never had any other outbreaks*.”

## 4. Discussion

In this paper, we share the experiences of 17 managers of ethnically focused senior homes in Canada during the COVID-19 pandemic. Qualitative analysis of the interviews resulted in four themes, which describe areas of impact and warrant attention and ensure that new policies from policymakers and other stakeholders take the perspectives of managers of ethnic senior homes in Canada into consideration.

### 4.1. Comparison with the Previous Literature

In the literature, it has been reported that other homes had similar challenges, such as homes for seniors of Japanese ethnicity, who nonetheless showed mental, physical, and social resilience in the face of adversity during the COVID-19 pandemic [21]. Further, the challenges of digital literacy and the need for support from agencies were observed in aspects of health, safety, connection, and belonging. A study of South Asian seniors in the Peel Region in Canada recommended a more proactive, inclusive, and people-centred (PIP) approach [22]. Our results back up the need for the PIP approach, where target areas in need of active policies and regulations are highlighted. A scoping review of experiences of racialized populations in Canada during the COVID-19 pandemic highlights inequitable experiences and indicates a need to address pre-existing inequalities and emerging discrimination [23]. Our results add the experiences and perspectives of managers, leaders, and decision-makers during the COVID-19 pandemic to the existing literature. Li et al. highlight the disproportionate increase in deaths in elderly nursing homes in the U.S. due to COVID-19 infections [24]. Our study neither solicited nor reported the relative impact of COVID illnesses and deaths in the homes.

### 4.2. Lessons for LTC Homes

The COVID-19 pandemic tested the preparedness of health systems across the world. Providing physical and mental health support was challenging due to additional burdens on the healthcare system and due to the healthcare workforce being redirected to essential services only. Surgeries and chronic illness treatments were halted, and consultations were limited to teleconsultations only with long waitlist times. With an ageing population suffering from dementia, assessing the psychological impact of the COVID-19 pandemic on LTC residents is difficult. The COVID-19 pandemic highlighted the need to address mental health and physical health support in combating social isolation. The period of the COVID-19 pandemic was understandably a period of emotional upheaval and had a significant impact on residents and their family members at all LTC homes. Shortage of staff and volunteers during the COVID-19 pandemic posed major hurdles to running the LTC homes effectively.

The effect of the earlier strains of COVID-19 were often severe and resulted in multiple infections and were heavily covered in the media due to the number of senior residents who lost their lives in Canada. However, in our interviews, we neither solicited nor reported deaths due to COVID-19 virus, as this was outside the scope of this study and such reporting could also have resulted in identification of our interviewees. Safety issues during the COVID-19 pandemic mainly revolved around infrastructure challenges, vaccine distribution, and ensuring enough personal protective equipment. Funding shortages made it difficult to implement the safety measures mandated by the government of Ontario. The detailed impact of political barriers and funding challenges and involvement of agencies cannot be understated. Involvement of agencies is critical to ensure the successful operation of any LTC, and it is important to address shortages in service delivery that were caused by financial barriers.

### 4.3. Lessons for Ethnicity Focused LTC Homes

Restrictions and bans on visiting communal places such as grocery stores, places of religious worship, or large gatherings exacerbated the social isolation experienced by the residents. While virtual programming and electronic and telephone communication were expanded, more mental, psychological, and social support was warranted. The COVID-19 pandemic necessitated rapid adaptation of alternate measures to cater to ethnic minority LTC home residents. From cultural programming requiring inclusivity to the celebration of festivals to applying safety measures to resident food services, creativity was employed due to the care and commitment of staff and facilities, despite funding constraints. Moreover, the challenges of visitor bans and a shortage of same-language-speaking staff contributed to challenges to residents’ emotional state in LTC homes specifically. Vaccine uptake increased due to trusted COVID champions explaining the benefits of vaccines to their respective communities.

Resource constraints need to be addressed to efficiently run ethnicity-focused senior homes. Limited resources can be mapped in various dimensions, such as the closure of LTC homes, a lack of ethnically focused LTC homes, and availability of staff with interpretation and translation skills, while working with the community where an ethnic home is located and navigating evolving technology to meet the needs of residents and their families. Staffing shortages and hiring issues related to language barriers (which were especially noticed among ethnic LTC homes), as well as hiring issues due to high turnover rates among the staff, were seen in the volunteer teams too. Volunteers have been a key part in managing operational expenditures with funding shortages, as they provided programming in residents’ native languages (a benefit of having volunteers in ethnic LTC homes) and helped combat social isolation through interaction and interpretation for staff and residents.

### 4.4. Implications and Future Steps

Nursing homes have to adapt in light of COVID-19, but there are particular circumstances and challenges related to ethnic minority populations that need to be considered given Canada’s growing ageing and diverse population. The COVID-19 pandemic necessitated creativity in terms of programming for residents of long-term care homes. Greater attention must be placed on virtual programming as well as assisting, educating, and familiarizing residents and staff with evolving technologies. Virtual community engagement will prove to be key in future pandemics. The International Agreement on Pandemic Prevention, Preparedness, and Response emphasizes focusing on vulnerable populations such as the elderly and those with chronic illnesses and indicates member states must have a comprehensive pandemic preparedness plan. It also demands that appropriate infection control and prevention measures be taken for long-term care facilities [25]. Focusing on the elderly and long-term care homes in terms of pandemic preparedness and response for future pandemics is key, and this study highlights the challenges, feasibility, and opportunities in this sector. Staffing is more difficult during times of crisis for these homes. People who are already isolated with dementia are more vulnerable in average senior homes. Provision of funding for additional costs and ensuring mitigation measures makes this more challenging. Extending funding packages at the federal or municipal level becomes crucial to ensure funding distribution to large immigrant populations and demographics where the multiethnic population is ageing.

### 4.5. Limitations

This study involved interviews with 17 leaders in ethnic senior care in Ontario, primarily in the GTA. As participation in the research study depended on availability and interest, personnel in the homes most affected might be represented, though we did have some participation, and those speaking appeared to benefit from a chance to escape day-to-day challenges and debrief. Further, using a snowball process may also have limited the spectrum of respondees. This study can benefit from interviews and subsequent analysis of residents and their families, policymakers, and other stakeholders to understand ethnic senior resident homes through a dual top-down and bottom-up approach. Caution needs to be taken regarding the research needs, as it has been less than five years since the COVID-19 pandemic at the moment of this study and the writing of this paper, and more long-term structural challenges and needs for improvement may be reflected in follow-up interviews with the interviewees.

## 5. Conclusions

In this study we have tried to reflect the experiences of the managers of ethnicity-focused senior resident homes in Canada during the COVID-19 pandemic. Through this study, we highlight that the ageing population requires that proper care, especially for the seniors facing dementia, be addressed in a culturally appropriate way and requires the extra measures of additional staffing and funding to ensure that proper protective measures are in place for ethnic senior care homes during periods of infectious diseases in Ontario, Canada.

## Data Availability

The datasets presented in this article are not readily available due to participant confidentiality and organizational anonymity. As per the protocol approved by the REB, the recordings and the raw data were deleted. De-identified and relevant quotations that support the study’s findings are provided in the Supplementary File S1. Requests regarding the datasets should be addressed to narya@uwaterloo.ca.

## Supplementary Materials

The following supporting information can be downloaded at https://www.mdpi.com/article/10.3390/ijerph23020152/s1: File S1: Raw quotes from the interviewees on their experiences of running a long-term care home during the COVID-19 pandemic in Ontario, Canada.
