# Supplementary material for: Operating Ethnicity-Focused Senior Long-Term Care Homes in Ontario, Canada During the COVID-19 Pandemic
Source: Int J Environ Res Public Health. 2026 Jan 26;23(2):152. doi: 10.3390/ijerph23020152 (PMC12940726; doi:10.3390/ijerph23020152)
Supplement: Supplementary file 1 [file ijerph-23-00152-s001.zip › ijerph-4028057-supplementary.pdf]

Supplementary File S1. Raw quotes from the interviewees on their experiences of running a long-term care (LTC) home during the COVID-19 pandemic in Ontario, Canada.

Pandemic management- Internal and External management			
Code Name<br>+ Definition (if needed)	Descriptor	Verbatim Quotes from participants	Document coding page number with Participant IDs included

Challenges	Pandemic challenges - health	A lot because of the additional practices that we've put in place, and, all those infection control, things like that. I think one of the biggest effects that COVID has had that we really cannot measure is the mental effects and the psychological effects that have affected the seniors because they can't, couldn't have their family members here. Like visit, because often, it is the family that is their kind of lifeline for the language and everything. So, from that perspective, it was, it was least for the residents who were cognitively aware and could appreciate that there's been a change and it's very hard to determine for the residents who are cognitively impaired, what, what they were really feeling and experiencing because it's very hard to really make that determination.	#008 Pg 59
	Pandemic challenges - Lack of volunteers.	All our histories of volunteers have done a great service. By the way, even though we were small. Before the pandemic, we used to have over 100 volunteers who would help with the recreational programs with feeding and so forth. And but right now, we're been struggling after the pandemic, people are still fearful of COVID.	#017 Pg 138
Staffing shortage -	Pandemic - hired staff not familiar with client language	during the pandemic we'd have had to hire can't be as picky with the languages and backgrounds of our workers just because of the Health Human resource shortages. And that did cause a concern among our clients. They were making requests. They wanted to be served by a certain type of nurse or social worker. And when we couldn't match it, there was noticeable distress.	#013 Pg 100
	Pandemic - difficulty in staff hiring	Yeah, for sure. We all know that it's hard to hire nurses now. So again, you can, you can't be choosers anymore, basically, employees market, if they want to work here they will. But if they want to change the scenery, they'll leave. So obviously, turnover is a big problem. So, when we hire quick, and sometimes we're not able to hire someone with the right background to serve our residents.	#013 Pg 102

	Pandemic - high death rate and new staff training challenges	COVID-19 was very devastating for this facility. We lost a lot of people in the first year, recovered well in the second year, but immediately we had to close down all these wonderful things I just spoke about has to be closed down, halls got closed down, services got shut down. And, and of course, there's one senior who were in the long-term care segments were felt very isolated. We've come out of that now, in a big way, you won't see a Senior with a mask on in this place, they've become kind of rebels. So, the shift back has been really, really good. No inhibitions at all about the shift back. But it was it was a tremendous time, a tremendous time for our staff, tremendous time for our seniors. And in the long-term care, we weren’t really heavily impacted. So, because we're in [large city], a lot of staff got COVID. So, we had to bring in a lot of agency staff and you bring an agency staff, it means it's people they don't know, they're not familiar with them. And that was its own challenges. So, our challenges for sure. But through it all, we were able to build a new long term care home, open it and move a whole bunch of residents into new long term care home. So that was amazing that I think they were actually able to do that.	#015 Pg 122
	Pandemic challenges - staff shortage and lost volunteers.	While it affected us and still is affecting us, because first of all, we lost almost all of our volunteers. And then we've been short staffed ever since, it's a constant challenge even. We have people that come in, and they will, if they don't like what's going on, or they don't think they get offered more money elsewhere, especially for that they've got to work for an agency because they can pick and choose their locations and the days they want to work and make their own schedule, basically following the line from us.	#017 Pg 141-142

Social isolation -	Challenges and Pandemic for Easy Access - Isolation	I would say that the covid 19 is definitely the main issue at this time. So, everybody needs to be safe and protect themselves, protect the residents. What I will say there will be much influence in terms of providing care through a telephone, but as well as on an online platform. But when you're on site, you need to make sure that everybody is healthy. Right. so that is very important. and then I'm thinking about the isolation piece as in like social isolation. cause we know that social isolation is a very important issue in the older adult population. And especially around this time, they can go anywhere, well not even grocery shopping as in like not recommended. And they can't go to community centers, and things like that. And for long-term care residents, they can have their family members come to visit them, only is like, as in like essential visit, as in like they're maybe at the end stage of their life. Maybe they're in palliative care, then their family members can come visit them as essential visit. So, during this time, I would say that the emotional support as in, as well as the mental support would be very critical. And I'm just wondering how we can do more in those areas	#004 Pg 31
	Pandemic - visitation challenges	Well part of the visitation, certainly because of the COVID. I mean, at one point, we weren't allowing family givers to come in at all. So, and then it was an hour that essential caregivers can come in, so have generalized unrestricted visitation. So again, they're not seeing all their family members. Maybe they had kids that dropped in once a month, but they probably haven't seen some family members, just because of those types of restrictions. I think that continues to be a really big one.	#008 Pg 59

	Pandemic - cultural difference on connectivity vs isolation	Well, regardless of color, race, or culture, everybody suffered from the pandemic induced isolation, separation, keeping an institutional setting that, as a given, might be seen through different lenses from particular cultural groups, depending on how much aid depends on that connectivity with the extended family. So that may be a cultural group specific. And again, acknowledging the risk of stereotyping. North American, or North American western culture, generally do not put as much new value, but they do not put as much emphasis on multi-generational conductivity. They value the visit by children or grandchildren. But they do expect children to grow and live their own lives. And then the older person, as much as possible, has their own independence and chooses their own life, even when they go into long term care. But the expectation of some minority cultural groups would be very much in terms of filial piety in the sense that there is a respectful elder and that they are not to be abandoned by the younger generation. So, they would expect to enforce separation isolation, perhaps a bit more acutely.	#011 Pg 90
	Pandemic - modifications for isolation requirements	the wings have been made into an isolation wing. So again, that's just to further speak to the OS not being able to offer optimal user space. Unwillingness now, or was previously during the peak of it all, just used to isolate. And then once they're done isolating began moving into an actual room. But the isolation rooms were once rooms themselves. So now we can't work capacity is lowered? What else? What? No, but let aside from that. I think that's the biggest impact as far as space goes.	#013 Pg 102

	Pandemic - number of beds in rooms and social distancing challenges.	Well, the one thing that COVID affected us, while you're well aware of this is we used to have two, four bed board. So, we can only have two beds in those rooms anymore. But fortunately, we had enough. For the first 20 years or so, we had the rest of bed, so we had extra rooms and beds there anyway, so we didn't lose any beds, it's just that we can't have the four bed wards anymore. And like I say, funding is the biggest challenge. People are happy.	#017 Pg 142
Limited resources	Pandemic and feasibility - challenges of getting closed but the push towards state of art facility and number of beds available and Benefits - of not having started facility during COVID and ability to build better	Well, except for now, now we will, we were one of the first to close because we recognize that we've become a vector for illness. Happily, no staff or client became ill. But the fact that these people were already compromised and, working in these areas was just, just mind boggling. but we were able to shut down. So, what we are aiming to do is build one of the largest long-term cares in the province in Mississauga. And unfortunately, we're having a hard time finding land. Fortunately, one of the silver linings I think will come out of COVID is a push to build state-of-the-art , infection, controllable long term care. We were happy that we were recognized as a culturally appropriate provider of care that people started to understand that we're the leading provider and that we would be able to, under contract support 128 South Asian beds. We've also put an application in for 220 beds in [large city] to build our own home where we'd be the lead as opposed to I don't want to say junior partner, but when you're talking to a hospital, everyone's smaller. So, the junior department. Yeah. So, we're feeling good about that	#001 Pg 6-7

	Pandemic for easy access and the challenges	Yeah. And, because it's been dynamic and then sometimes people get sick or they need to be quarantined. So, they would definitely, we'll have, less staffing during this time. So, limited resources, but definitely lots of things that we need to be to do would be a huge challenge. And to think creatively as well as in like as you like, we can provide care well as in like community care in-person care. People can't come to the community center to participate in programs. Then how can we think creatively to address their needs and to continue doing, supporting them would be something that we need to think about as well.	#004 Pg 33
	Pandemic - religious stuff suspended	The religious stuff is suspended. So, that's, there's, there's an exhaustive list that I'm, that isn't at my fingertips. What's the best way for me to get that to you? So, the answer to your question is yes, like emphatically yes. and these are all the building blocks of a culturally appropriate experience. Yeah, they're essential. but in order to get you the, the, the accurate list. And then I don't know if it's interesting to you, but like, which of those have been modified or suspended because of the pandemic? That information I would have to get to you, but what, like what's the best way to, well, first of all, how much detail do you want? Like I can set you up to talk to someone and they'll overwhelm you with the complexity with the breadth of our program. But what are you actually looking for? What's a good source?	#006 Pg 47-48

	Pandemic - understanding of fairness	Well, I'm sorry, I don't have a crystal that whether you're talking about cultural specificity, or ageism, or whatever it is talk about in healthcare is and perhaps it reflects my professional background, individuation and personalization and client sentiments is always to go by, whether you're talking about working with a particular community or cultural group, then what is your understanding of the cultural group? How are you going to leverage the strength of that particular ethnic cultural group and their values and evolve your way of working with them? Understanding that multiculturalism is an ideal state, it does not guarantee that your whole facility, your whole staff group or the rest of your clients, safe facility, shares in that viewpoint in the same way, and so be careful about what would be seen as favoritism to particular groups. What is fair and accommodating for those with?	#011 Pg 90
	Pandemic - additional expenses and financial constraints	Financially it affected us because we just spray the place twice a day. We've tried to make sure that they wear a mask, we got their vaccines for them. Because of the second vaccine, we got a third backseat. So, I think, and oh restricted visitors. That was the main thing, I think, is the visitors who bring in the COVID more than anything else. And that was where these old people got it. Of course, they're more susceptible to COVID more than other people.	#012 Pg 98

	Pandemic - communication challenges to provide proper information	Well, I think that COVID-19 has impacted hard need to really increase our communication. So, we're needing to ensure that all of our people living in our homes have the proper communication. We primarily deliver our communications in English at the corporate level, but then when it comes time to actually talking to the residents or family members. They can the person's specific language to help them understand what is happening. And so, we've had to be really mindful of communication and what everyone needs in order to receive the proper information and ensure that everyone is well.	#014 Pg 105

	Pandemic and evaluation - funding challenges and working with the agency	Just by I mean, when I six months I call the office. So, when I had an issue with the Ministry of have given us a rough time on the COVID funding, which is a big secret, people don't know that they pretend to have funded COVID. But they don't give you the money to like a more than it's like a year and a half later. I had to make some calls about that. Because I said, Come on, we've got to pay our bills. And we've got this receivable that the Ministry of Health has put forth, and I've given us money for first person I call was Patrick Brown. And they organized a whole bunch of meetings, their responses office, we are a guest, we invite them every time we have a celebration, we open like that new long term care home. And I mentioned that opened during COVID. They came I mean, he's been here many times personally, to do ribbon cuttings and speeches. And we have the guttural family here a lot. And we always invite the mayor's office and they always come. So that really pre-existed before I got here. But it was pretty easy to maintain. Once you make your phone call and say I'm calling from Holland Christian homes, they were like, Yeah, that's great. So it's important to have that relationship for them to know that they've got a major see is complex in their writings. So and also, I think we are the writing for the Secretary yet. So, you have to know your MPP is as well and, and just call them and then once you call them, they are responsive. They've come here to do talks, and you've got to make sure you invite them for celebrations as well, not just when you have concerns or complaints.	#015 Pg 125

	Pandemic - technology challenges	In some ways, that is interesting question, because in many ways, my world did not change throughout the pandemic, from beginning to end, we still continue to provide support in people's homes, where possible, remote far the population base that I work with, is by phone, right, they don't have access to internet, or technology, many of my clients don't even have a phone. Largely because especially for my clients who are, let's say, living in market rent, their entire income goes to rent, leaving them sometimes very little, like maybe 100, or $200. And so, they are getting food to the mobile food delivery. The pandemic really impacted them in huge ways. So, the only change that really took place was having to facilitate virtual appointments. Because if they didn't have a book, if they don't have a phone, they don't have a smartphone, they don't know how to use the technology from the virtual zoom type of platform, which many physicians and specialists, especially the specialists in the hospitals. And so, a lot of my home visits and home visits, became and continue to be that like yesterday, I had to facilitate a virtual speech language pathology appointment for a client in their home. So also, with one of your colleagues, ODSP, or any of the government systems that are out there, everything's moved to a virtual platform. So, it left an entire demographic out.	#016 Pg 127
	Pandemic and challenges - technology and phone usage is difficult and busy lines	Absolutely, where, of course, a lot has changed them, where like, they had a caseworker, and now it's sort of even, for example, Public Guardian trustee. Clients would have their trustees phone number, now, it's all a call center. And it takes forever. And, many of my clients don't have the capacity to navigate. A simple phone call that layered, right. And so, yes, we've had to pivot to do a lot more hand holding when it comes to system navigation.	#016 Pg 128

	Pandemic - Preparedness and Stakeholder and policymaker interests	While or that's the thing, we're in the process of preparing for, whether it's anything like this or any other diseases. The impact is, is a big thing now with the Ministry of Health and Long-Term Care. And we were working with our Ontario Health team and we're working very closely with the Sunnybrook Hospital and on our infection control practices and everything and a lot of emphasis on that so, right now everybody gets screened and gets tested and so forth.	#017 Pg 142
Infections	Pandemic - outbreak experience	So, COVID, because we've established that community, we didn't have any problems during COVID time. We just treat everyone the same way. And, we've never had other than one outbreak with one resident that made it, die of it. And it was actually a year, we've never had any, like other outbreaks.	#010 Pg 79
	Pandemic - deaths due to COVID. Post vaccine less serious illness.	unfortunately, we had about [number of] deaths due to COVID. And in the beginning, before the vaccines and everything sort of spread, like wildfire. And even, we had a couple of residents that were even bedridden, and they still got COVID. But, ever since the vaccines and everything like that, we've had some cases, but they don't get serious illnesses like they did in the beginning of the pandemic.	#017 Pg 142

Safety	Pandemic - safety - perception of long-term care homes and challenges.	Your question on 28th of April isn't separable from the, from a global pandemic. So right now I think the entire community, I think anybody with a senior in their lives is terrified, like mortally afraid of what the pandemic is doing and long-term care homes happen to be more vulnerable than, than other environments. I'm sure you're seeing the news coverage of what happens when, when COVID 19 gets into a long-term care home and it's terrifying. I mean this is what we talked about at the very start of our conversations. This has to be the first thought that people in any community land on when my long-term care comes up. In fact, for some of these, I can't recall why I was doing this, but I was trying to Google long-term care for some purpose, completely unrelated to pandemic. And of course, as soon as you do that, it's like, it's all about like death and, and terrifying things come up. Like the first things that, that auto populate in Google when you type in long-term care, it's terrifying stuff.	#006 Pg 45
	Benefits - safety in LTC homes	long term care is very tightly regulated in Ontario, so I'm going to guess that safety is the same.	#006 Pg 42
	Pandemic - perception of people and danger	yes, I think that's what preoccupies people right now. It's the danger that pandemic poses to frail seniors in group living environments.	#006 Pg 46

	Pandemic - infrastructure challenges	What, I really don't know what we can change because of our physical structure and layout. We don't have the space to do a lot of things. To build a new building, it would be nice, because then we could incorporate all kinds of things into the new building that would make handling a pandemic, I would think, not easy, but certainly we would be able to manage it in a different manner. Because we are a single unit, it creates issues, learn how to, I don't like using the word isolate people, but , to have the residents, to be able to mix and mingle and have some of their programs and things like that, because we just don't have that space to accommodate those needs.	#008 Pg 60
	Pandemic - infrastructure challenges	That's right, and we just don't have that physical space to look at doing anything like that. So, I mean, for our residents, when the pandemic first started, they were basically and I mean, without a word of a lie, they were basically confined to their rooms. There was no way that that we could even bring them out into a lounge area in small groups to at least have a change of scenery or something. That was just not possible to do. And because we're an older building, we don't have the airflow that would be needed to help sort of keep various sections so that the air isn't intermixing and what I mean. So that, that is a big challenge. And that challenge can never be overcome, until we actually have a brand new physical structure.	#008 Pg 60
	Pandemic - mask wearing of staff	But we are still all wearing. Like all the staff are wearing masks. The residents aren't, but the staff are. And then of course, if they go on to medical appointments, their, their, their follow up and that sort of thing so. So staff and residents are having more social interaction. So controlled.	#008 Pg 60

	Pandemic - vaccine distribution and decision by family members, individual centred organization, empowerment	we did over 400,000 vaccines for seniors in East Toronto. And it was massive. So, bringing them down having calling their daughters, for some of them, like, I don't make any decisions only my daughter does, or all sorts of things. Like, it's just you have got to the biggest thing we've learned from COVID, is you've got to meet people where they're at, not where you think they should be?	#009 Pg 64
	Pandemic and Evaluation- infrastructure expectations and health screening	Well, I like I don't think I need to tell you that if you look at the long term care applications, they will tell you specifically what they expect, if you want to look for a highly regulated industry with long term care, they'll tell you everything, you're gonna have to have isolation rooms, gonna be able to monitor sort of cross pollination of people, all of that screening all of that we'll be able to.	#009 Pg 65
	Pandemic - clients safety	I think they all were vaccinated, one or two had COVID, but that many. We were we were very lucky that way. About four, about four people had COVID as far as I know, there were no mortality from it. No morbidity as well as no post COVID morbidity, like chronic lung disease or anything like that so we were lucky [inaudible]	#012 Pg 97
	Pandemic - increased expenditure and Personal Protective Equipment (PPE) costs	everything's more costly, because, for example, shared bathrooms, like no, we have to avoid sharing. We have to avoid admitting patients into rooms that they have to share with others. Just to keep them as best as we can. And Trooper rooms shoe for bathroom shoe for everything. So, we can't optimally use our space PPE all that stuff. Big, big impact on cost. And there's so much more mainly using space. We're we're constructing our long-term care home in Richmond Hill now and we've had to redesign it a little bit to meet the needs of the new landscape.	#013 Pg 101

	Pandemic - safety measures	Yeah, it's hard for me to comment on that. But I know that we've had a really good safety record. I don't know if it's because of cultural appropriateness, or just that, we're really diligence. But, certainly, having a close relationship with our resident clients and family members helped. And our staff helps everyone to stay safe. So because we move together as almost like an extended families, and people are more cooperative, right, and they're more understanding, and they're willing to, for example, get the COVID vaccine, no matter how many vaccines there are, people are willing to, hear us and, listen to us always say, it's probably a good idea that you get the fourth vaccine, so people are more receptive, to doing what we ask, when we ask people and family members to wear masks, they're more inclined to cooperate because we have a tight knit relationship. So that all contributes to safety, right, the hand hygiene and asking the vaccinations and when we have, and new situations come up, they're very understanding, and we communicate with them quickly, and we resolve their questions. So, it's just, that's why we're able to create that environmental safety.	#014 Pg 113
	Pandemic - preparations in terms of PPE	what's the biggest challenge was the fact that we are at our staff within [large city], it's very dead serious, so they get sick. And so, all I can do with that really is you PPE, but people are going home to kids who are coming from school. And, there's not a whole lot you could do about the spread of the disease outside of here. Like when they're in here. They're from full gear. When they go home. They're not. So, I don't think there's anything more we could do. I mean, we We're doing the maximum we love beyond we've got above public health requirements in terms of testing, and rapid testing, and I'm checking on people. So that's really all you can do.	#015 Pg 122

Emotional Impact	Pandemic challenges - family concerns	what else is making it complicated for families that have a senior in care. So the very first thing that occurred, like when the when the pandemic became a local and real, you remember in, in the middle of March when all that starts happening. And like, I mean, just as a citizen, it made my head spin like what, what's happening? Like we were prepared for the pandemic and had been preparing for the pandemic for months. In fact, it's part of our normal operations to prepare for a pandemic. We were preparing for this pandemic intensively since December, but then in, in March when sort of the whole world went weird. Like when, when nothing was familiar. The very first thing that our, that our families would have seen was a visitor ban and that was an essential step to keep the facilities safe. I don't contest the ministry decision to do that, but what it meant was at exactly the moment when our families most vulnerable, the curtain came down and, and it was no longer possible to see what was going on inside. So like whatever if you mapped this to your own experience, whatever feelings of vulnerability or compassion or worry that you had for your own elders in your own home, those feelings would have to be magnified by this veil that came down between you and your loved one inside a locked facility. And I can only imagine these feelings cause I'm not in that circumstance, but I deduce that our families were and are terrified for the safety of their elders, the elders living in our long-term care facilities. So, I mean on one hand, like there is risk long-term care homes are under siege by the virus right now. And then on top of that, no one can see what's going on. Like no one, no one can go in and, and touch their grandma or no one can give their grandpa hug. They can't. It's just, it's not this crazy convergence of, of awful circumstances. Means that people are at least able to address their most pressing concerns.	#006 Pg 46-47

	Pandemic and benefits - importance of champions - COVID as an opportunity mobilizing for vaccines	So, for example, when I think about the work that we did with COVID, right, and getting a lot of these communities vaccinated, because there was a lot of fear, and they couldn't get out of their homes, they couldn't line up, etc. Part of what we had to do was to translate the information for each of the different communities. But we also then had to find champions in those communities that can help to be ambassadors for that work, so really going to the source and understanding the cultural nuances are really important. So, for example, and I think about, the South Asians, like the Indian community, or the Muslim community that was in Thorncliffe, Park, and they would listen to their Imams, or, their religious leaders, they were the ones who could convince them in another community, it was somebody else. So, I think you have to, and I also worked with a lot of faith-based organizations who were trying to do good work, but doing a horrible job of it, and being able to take because you're offering them this, but they actually don't want that. But they don't want somebody from their own community, because they don't want you to go and tell everyone their business and, all the rest of it. So, it's really championing your population. And working with those people that are leaders, but also allowing others that are trying to be leaders, how do you build their capacity to do more work?	#009 Pg 63-64

Mitigation	Pandemic - no additional funding but requirement to adapt to new cultural programming with pandemic restrictions	Well, especially during COVID-19, when we need to pivot so much changing our in person programs to virtual programs, communicating much more frequently. when people are isolated and can't celebrate Christmas or Easter or whatever holiday together, we're needing to ensure that the meals are extra special, right, that they can also celebrate, even though their loved ones aren't allowed to come and visit or if you're just living at home independently, you're not going out yourself. So, there's been all sorts of pressures on organizations not only limiting the home, but everywhere, including those who are culturally appropriate to really move fast and just continue to use existing resources without any additional funding to change our programming or to move as fast as we could	#014 Pg 108
Meal on wheels -	Pandemic - meals on wheels program	Cause like we still have the meals on wheel program.	#004 Pg 30
Telephone and online support groups -	Pandemic for easy access - long term care support online as well as over phone	I would say that staffing is always an important issue, especially doing like a pandemic. Like people may get sick. We definitely need people on site to support the long-term care as well as maybe some community program as well. We still have telephone as well as online support groups going on	#004 Pg 30

	Pandemic for easy access - Online support groups and telephone support	I was saying that during this time or the colleagues are being very creative, so we look into gaps and ways and how we can better support our clients as well as our residents. So, knowing that family members can’t come to visit the residents. So, we provide comfort calls as you like. We have a staff on site to call the families to tell them that the residents are fine and answer any question they may have. And we also support video chat so that we can facilitate video chat between resident as well as resident families’ members. And that's also applied to the community side, as in let's say the day program clients can come to our program at this time, but we provide comfort calls to them as well. So, we check in to see how they're doing, if they have any needs, if they need assistance or if they need any help, make referrals etc. As well as we provide telephone support groups and online support groups. So that people are connected, maybe one day everything is fine back to the new normal they say. So, we will welcome them to come back to our programs rather than just a very rough cutoff and there's no contact during this time. So, I do think that's very important as well.	#004 Pg 30-31

	Pandemic - setting up communications	We tried to address that in our communications and this is actually where most of my energy has been focused in the last six weeks. So, we've, I don't know if you had a chance to dig around on the [ethnic LTC] website. What you'll find is that our communications are tailored specifically towards disclosure. So, you can't be here to touch mom or to go give grandpa a hug. But we'll tell you everything that we can, we'll tell you everything you need to know about what's going on in here. There's lots of photos. There's like, we've got a whole good news page on our website, which is just about making more visible what it is that we're doing. If we weren't doing that, we know that social media would go bananas with all kinds of theories about what was going on inside, and facilities. But we can proactively address some of that, some of that worry and some of that interest, by just showing what we're up to.	#006 Pg 47
